# Incidence of Breast, Prostate, Testicular, and Thyroid Cancer in Italian Contaminated Sites with Presence of Substances with Endocrine Disrupting Properties

**DOI:** 10.3390/ijerph14040355

**Published:** 2017-03-29

**Authors:** Marta Benedetti, Amerigo Zona, Eleonora Beccaloni, Mario Carere, Pietro Comba

**Affiliations:** Department of Environment and Health, Istituto Superiore di Sanità, Viale Regina Elena 299, 00161 Rome, Italy; amerigo.zona@iss.it (A.Z.); eleonora.beccaloni@iss.it (E.B.); mario.carere@iss.it (M.C.); pietro.comba@iss.it (P.C.)

**Keywords:** cancer, incidence, endocrine disruptors, environmental exposure

## Abstract

The aim of the present study was to investigate the incidence of breast (females), prostate, testicular, and thyroid cancer in the Italian National Priority Contaminated Sites (NPCSs), served by cancer registries, where the presence of endocrine disruptors (EDs), reported to be linked to these tumours, was documented. Evidence of carcinogenicity of EDs present in NPCSs was assessed based on evaluation by international scientific institutions and committees. Standardized Incidence Ratios (SIRs) were computed for each NPCS and cancer site between 1996 and 2005. Excess incidence of one or more cancer site studied was found in twelve out of fourteen NPCSs. Significantly increased SIRs were found for breast cancer in eight NPCSs, for prostate cancer in six, for thyroid cancer (both gender) in four, and for testicular cancer in two. Non-significantly increased SIRs were found in five NPCSs for testicular cancer and in two for thyroid cancer (males). In a small number of instances a significant deficit was reported, mainly for thyroid and prostate cancer. Although increased incidence of one or more cancer sites studied were found in several NPCSs, the ecological study design and the multifactorial aetiology of the considered tumours do not permit concluding causal links with environmental contamination. Regarding the observation of some excesses in SIRs, continuing epidemiological surveillance is warranted.

## 1. Introduction

Over the past decade, in industrialized countries served by cancer registries, an increased incidence of breast, prostate, testicular, and thyroid cancer has been observed [1,2]. The increase in incidence of endocrine-related cancers in humans cannot be explained solely in terms of genetics, better diagnosis, or life style. Currently, it is believed that the increase may be partially related to environmental chemical exposure, some of which have endocrine disrupting properties [2].

An endocrine disruptor (ED) is commonly defined as an exogenous substance or mixture that interferes with the production, release, transport, metabolism, binding, action, or elimination of natural hormones, and which consequently causes adverse health effects in an intact organism, or its progeny, or (sub)population [2,3]. Like hormones, EDs can act at low dose, may have non-monotonic dose responses, will have tissue specific and time effects, will show different effects and dose responses during development relative to adults, and are likely not to have a threshold [4].

Many chemicals have been identified as endocrine disruptors, and humans can be exposed to them either due to their occupation, through dietary and environmental exposure, or both [5]. Among environmental pollutants, the best-characterized chemicals with endocrine disrupting properties considered to be involved in cancer aetiology include dioxins, dioxin-like compounds, furans, polychlorinated biphenyls (PCBs), solvents, heavy metals, dichlorodiphenyltrichloroethane (DDT) and its metabolite dichorodiphenyldichloroethylene (DDE), as well as other pesticides [6,7].

By far the most studies relating to the association between EDs and tumours have been carried out for breast, prostate, and testicular cancer [8,9,10,11,12,13,14,15,16,17,18,19,20], while thyroid cancer has received very little attention. Because of concerns associated with the increase of endocrine-related cancers, several international scientific organizations and advisory committees have included among their priorities the need to implement epidemiological studies to integrate epidemiological data with data on the environment, food chain, and human biomonitoring data, and to strengthen studies on chemical mixtures exposure [21]. 

The aim of the present study was to investigate the incidence of breast, prostate, testicular, and thyroid cancer in the Italian National Priority Contaminated Sites (NPCSs), served by cancer registries, where the presence of EDs linked to these tumours was reported. The study was part of the Italian Epidemiological Study of Residents in National Contaminated Sites (SENTIERI Project) [22].

The Italian NPCSs are characterized by the presence of major industrial activities (refineries, petrochemical, and metallurgic plants), and industrial and uncontrolled waste sites. In these NPCSs, several environmental pollutants, some of which have recognized or suspected endocrine disrupting properties, were detected. PCBs, dioxins, heavy metals, and solvents represent the principal sources of pollution in these areas; in most of the NPCSs they occur in the form of mixtures. Combinations of EDs can produce a significant effect, even when each chemical is present at a low dose that individually does not induce observable effects [23,24].

Thyroid cancer was included even if the current understanding of its aetiology does not clearly link it to an endocrine mechanism. Still, some experimental and epidemiological studies have suggested that oestrogen may play an important role in the development and progression of papillary thyroid cancer; this might make it plausible that xenoestrogens, such as cadmium or 2,3,7,8-tetrachlorodibenzo-p-dioxin (TCDD), may also contribute to the risk [25,26].

It is worth noting that the aetiological role of EDs and other chemicals in the pathogenesis of endocrine tumours is still a matter of manifold research activities, and the mechanisms through which chemicals may induce these tumours are not completely understood. At present, researchers have mainly focused their attention on the disrupting properties of chemicals related to the pathogenesis of endocrine tumours. However, other mechanisms, such as oxidative stress and DNA damage, may potentially contribute to the carcinogenesis of endocrine tumours. For example, many heavy metals, dioxins, PCBs, polycyclic aromatic hydrocarbons (PAHs), and other carcinogens have been shown to cause oxidative stress [27,28,29]. Nevertheless, according to Silisin and Högberg [30], “oxidative stress sometimes can be an important causative factor, but sometimes only a bystander in an agent’s, or a mixture’s, toxicological profile”. Moreover, some EDs could act through an indirect mechanism, as is the case of breast cancer. For this tumour, several studies suggest that the bioaccumulation of some persistent organic pollutants (PCBs and TCDD) in adipose tissue promotes the development of obesity and ultimately either influences development or progression of breast cancer, or both [31].

## 2. Materials and Methods

This is an exploratory ecological study. Cancer incidence (all ages) was investigated for breasts (females), prostate, testis, and thyroid, based on figures produced by the Italian Association of Cancer Registries (AIRTum), within the SENTIERI Project [22], as discussed elsewhere [32]. Evidence of carcinogenicity of EDs for the tumours included in the study was assessed based on evaluations carried out by international scientific organizations and advisory committees. We took into consideration only the EDs that could be identified as environmental pollutants in the study areas. Relevant papers were identified from five major sources: World Health Organization/United Nation Environment Programme (2013), International Agency for Research on Cancer (2015), European Commission (2012), European Environment Agency (2012), and The Endocrine Society (2015) [2,33,34,35,36]. This was considered an accurate account of the state of science on the potential human carcinogenic health effects of environmental exposure to EDs up to a year prior to their publication. To update such evidence, relevant literature published in the last four years has also been reviewed. This literature search was carried out in PubMed. In addition, a search was carried out on available environmental data to ascertain the presence of EDs in the NPCSs in the study. The environmental data were collected from the Italian Ministry of Environment, from the legislative national decrees where the NPCSs are defined, and from local environmental agencies [37,38]. The available data are related to the presence of the substances in the various environmental matrices, not necessarily to their concentrations. The existence of human biomonitoring data (blood, serum, and milk) reporting high levels of endocrine disrupting chemicals and other monitoring data (food and plants) regarding the NPCSs was verified by a search in PubMed [39,40,41,42,43,44,45,46,47,48,49,50,51,52].

Age, gender, and socio-economic-deprivation-index adjusted Standardized Incidence Ratios (SIRs), with their 90% confidence intervals, were computed for each NPCS and cancer site by the Italian Association of Cancer Registries [32], referring to the 1996–2005 time window. Reference rates were derived from the pool of cancer registries from Northern-Central Italy or from Southern-Central Italy, depending on the study area localization. 

## 3. Results

Seventeen NPCSs served by a Cancer Registry were identified. Three NPCSs were excluded from the study, as the presence of EDs could be reasonably excluded. Therefore, the NPCSs included in the study were fourteen. 

The environmental pollutants with suspected or recognized endocrine disrupting properties reported to be carcinogenic for the tumours studied by the five abovementioned international scientific institutions/committees are shown in Table 1. Information on the characterization of the NPCSs with respect to the presence of major sources of pollution is reported in Table 2, together with the indication of the EDs of interest detected in the environmental matrices, human biomonitoring data, and food monitoring data. The age, gender, socio-economic-deprivation-index adjusted SIRs, with their 90% confidence intervals (90% CIs), are reported in Table 3. Excess incidence of one or more cancer sites studied was found in twelve out of the fourteen NPCSs. Significantly increased SIRs were found for breast cancer in eight NPCSs, for prostate cancer in six NPCSs, for thyroid cancer (both genders) in four NPCSs, and for testicular cancer in two NPCSs. Furthermore, non-significant increases in SIRs were found in five NPCSs for testicular cancer and in two for thyroid cancer (males). Significant SIRs deficits were also found: for thyroid cancer in four NPCSs (in one NPCS in both genders, in three NPCSs only in women), for testicular cancer in three NPCSs, and for breast cancer in one NPCS.

## 4. Discussion

A statistically significant excess in incidence occurred in twenty-two circumstances concerning twelve NPCSs, mainly for breast and prostate cancers, which are relatively common tumours. The low number of testicular and thyroid cancer excesses may be explained by the sparse number of cases on which the findings were based. In a smaller number of instances (eleven circumstances concerning six NPCSs) a significant deficit was reported, mainly for thyroid and prostate cancer. Excesses were altogether more frequent than deficits. These findings are suggestive of an overall adverse effect associated with residence in NPCSs, even if a role of random variability, confounding and alternative explanations cannot be ruled out, due to the adoption of an ecological study design. 

Additional research should consider the peculiarity of each of these tumours (different window of vulnerability, peak incidence, reported shift of the age of onset, and increase of specific histotypes). Due to the rarity of testicular and thyroid cancer, an extension of the length of study window is indicated.

## 5. Conclusions

Due to the ecological approach of the study, we could not adjust for several confounding factors reported to be risk factors or sources of exposure for the tumours studied (smoking, alcohol consumption, obesity, genetic predisposition, use of personal care products, and use of pharmaceutical drugs) [1,2]. Thus, even if an increased incidence of one or more cancer sites studied was found in several NPCSs, the study design and the multifactorial aetiology of the considered tumours do not permit conclusions to be formed in terms of causal links with environmental contamination. Moreover, the complexity of exposures to multiple chemicals with suspected endocrine disrupting properties, and the possibility of other potential mechanisms of their action (oxidative stress, or DNA damage) [27,28,29], make it difficult to hypothesize on substances and mechanisms that have determined the excesses of tumours in some NPCSs.

However, the observed increases encourage further analytical epidemiological studies to be performed. Due to the observation of some excesses in SIRs, chemicals reported or suspected to be carcinogenic for the tumours studied should be a priority in environmental clean-up, even if they do not act through endocrine-disrupting mechanisms [2,21,53,54]. Furthermore, a continuing epidemiological surveillance is warranted in the NPCSs. An exposure assessment that includes the detection of further emerging contaminants with endocrine disrupting properties, such as perfluoroalkyl substances (PFAS), is also recommended.

## Figures and Tables

**Table 1:** Environmental pollutants with endocrine disrupting properties considered to be carcinogenic by scientific institutions/advisory committees for the tumours studied.

Cancer Site	IARC [1]	WHO/UNEP [25]	European Commission [26]	European Environmental Agency [27]	The Endocrine Society [28]
Breast	PCB Ethylene oxide	Dioxins Furans PCBs Solvents	Cadmium Solvents	Oestrogenic EDs	Dioxins
Prostate	Arsenic Cadmium Rubber production industry	Arsenic Cadmium PCBs Pesticides	Arsenic Cadmium PCBs Pesticides	Pesticides	Cadmium Farming PCBs
Testis		Prenatal exposure to POPs Fungicides PBDE Pesticides	Organochlorine chemicals (including DDT and some pesticides) PCBs	DDE DDT PCBs	Arsenic Cadmium PCBs
Thyroid		Pesticides TCDD	PCBs Pesticides Solvents	PCBs	

DDE: Dichorodiphenyldichloroethylene; DDT: Dichlorodiphenyltrichloroethane; PBDE: Polybrominated Diphenyl Ethers; IARC: International Agency for the Research on Cancer; PCBs: Polychlorinated Biphenyls; POPs: Persistent organic pollutants; TCDD: 2,3,7,8-Tetrachlorodibenzo-p-dioxin; WHO/UNEP: World Health Organization/United Nations Environment Programme.

**Table 2:** National Priority Contaminated sites (NPCSs) information on pollution sources, and endocrine disruptors (EDs) of interest detected in environmental matrices, human biological samples, and food.

NPCS	Area Description		Other Data on EDs of Interest	
	Pollution Sources	EDs of Interest Detected in Environmental Matrices	Human Biomonitoring	Food
Bacino Chienti	Shoe factories	PCDDs/PCDFs, benzene, toluene, other solvents		
Brescia Caffaro	Chemical plants, landfill	As, PCBs, PCDDs/PCDFs, chlorobenzene	PCDDs/PCDFs, PCB (human serum)	PCB (food of animal and vegetal origin); PCDDs/PCDFs, PCB (cattle’s meat, cow milk, forage)
Fidenza	Chemical plants, urban and hazardous waste landfills	As, PCBs, PCDDs, benzene, other solvents		
Litorale Domizio Flegreo	Urban waste landfill, illegal dumping sites, illegal burning of waste	As, PCBs, PCDDs, benzene, other solvents	PCDDs/PCDFs (breast milk)	PCDDs/PCDFs, (cow and buffalo’s milk)
Laguna Grado Marano	Cellulose production plant, dockyard	As, PCDDs, benzene, other solvents		
Laghi Mantova	Metallurgy plants, paper plant, petrochemical plant, harbour area, industrial waste landfills, hazardous waste incinerator	As, Cd, PCDDs, ethylbenzene, other solvents		PCBs (fruit, vegetables)
Milazzo	Oil refinery, steel plant, thermal power plant, electrical equipment factories, illegal dumping site	PCDDs, heavy metals. Benzo(a)pyrene	Cd, As (serum)	
Porto Torres	Chemical plants, petrochemical plant, refinery, power plant, harbour area, illegal dumping site	As, Cd, chlorobenzene, other solvents		PCDDs (fish and other seafood)
Priolo	Chemical plants, petrochemical plant, refinery, harbour area, hazardous waste landfills	PCB, hexachlorobenzene	Dioxins, PCB, HCB (breast milk, and puerperal hair)	Cd, Pb, Hg, PCDDs, organochlorine compounds (fish and other seafood)
Sassuolo-Scandiano	Ceramic industries, industrial waste landfills	Heavy metals		
Taranto	Oil refinery, steel plant, harbour area, cement plant, controlled and illegal waste dumps	As, Cd, PCDDs, PCBs, benzene, xylene	As, Cd (serum and urine); PCDDs, PCBs (serum and milk)	PCDDs, PCB (sheep and cow’s milk, clams); PCB, HCB, PAHs (clams)
Terni-Papigno	Steel plant, hazardous waste landfills	PCB		
Trento Nord	Chemical plant	Solvents		
Venezia Porto Marghera	Chemical plants, petrochemical plant, oil refinery, harbour area, illegal dumping sites	As, Cd, PCBs, PCDDs, solvents		As, Cd, PCDDs, PCDFs (shellfish)

NPCS: National Priority Contaminated site; As: Arsenic; Cd: Cadmium; EDs: Endocrine disruptors HCB: Hexachlorobenzene; PAHs: Polycyclic Aromatic Hydrocarbons; PCDDs: Polychlorinated dibenzo-p-dioxins; PCDFs: Polychlorinated dibenzofurans.

**Table 3:** Standardized Incidence Ratios (SIRs) with 90% confidence intervals (CIs), 1996–2005.

NPCS (Geographical Area)	Thyroid Cancer				Testicular Cancer		Prostate Cancer		Breast Cancer	
	Males		Females						Females	
	obs.	SIR * (90% CI)	obs.	SIR * (90% CI)	obs.	SIR * (90% CI)	obs.	SIR * (90% CI)	obs.	SIR * (90% CI)
Basso Bacino Fiume Chienti (Central Italy)	6	83 (36–163)	21	85 (57–122)	11	148 (83–245)	181	120 (106–136)	227	117 (104–130)
Brescia Caffaro (Northern Italy)	47	170 (132–217)	131	156 (134–180)	31	102 (74–137)	807	124 (117–132)	1187	125 (120–132)
Fidenza (Northern Italy)	18	145 (94–215)	32	88 (64–118)	15	134 (83–207)	339	105 (96–115)	403	102 (94–111)
Litorale Domizio Flegreo and Agro Aversano (Southern Italy)	54	95 (75–119)	147	69 (60–79)	70	108 (87–131)	404	76 (70–83)	1097	103 (98–108)
Laguna Grado Marano (Northern Italy)	3	33 (9–86)	15	57 (35–88)	15	176 (109–272)	216	107 (96–120)	249	95 (85–106)
Laghi Mantova (Northern Italy)	21	174 (117–251)	58	155 (123–193)	17	141 (90–211)	315	103 (94–114)	472	113 (105–122)
Milazzo (Southern Italy)	6	124 (54–245)	24	140 (96–196)	4	98 (34–225)	54	99 (78–125)	80	108 (89–130)
Porto Torres (Southern Italy)	30	69 (50–94)	155	97 (84–111)	51	135 (105–170)	601	137 (128–147)	966	125 (119–132)
Priolo (Southern Italy)	34	89 (66–119)	132	94 (81–109)	37	103 (77–136)	417	105 (96–114)	712	111 (104–118)
Sassuolo Scandiano (Northern Italy)	41	146 (111–190)	106	130 (110–152)	39	121 (91–159)	540	92 (86–99)	702	90 (85–96)
Taranto (Southern Italy)	34	158 (116–210)	98	120 (101–142)	20	108 (72–158)	303	130 (118–143)	497	145 (134–156)
Terni Papigno (Central Italy)	32	106 (77–142)	67	66 (53–81)	32	121 (88–163)	577	89 (83–95)	902	114 (107–120)
Trento Nord (Northern Italy)	20	71 (47–103)	71	70 (57–85)	32	104 (76–140)	527	88 (82–94)	876	98 (92–103)
Venezia Porto Marghera (Northern Italy)	57	74 (59–92)	165	71 (62–81)	76	94 (77–114)	2075	103 (100–107)	3045	110 (107–114)

*: adjusted for age and socio-economic deprivation index; Abbreviation: obs.: observed cases.

## Abbreviations

The following abbreviations are used in this manuscript:

As	Arsenic
Cd	Cadmium
CI	Confidence Interval
DDE	Dichorodiphenyldichloroethylene
DDT	Dichlorodiphenyltrichloroethane
EDs	Endocrine disruptors
IARC	International Agency for Research on cancer
HCB	Hexachlorobenzene
NPCSs	National Priority Contaminated Sites
PAHs	Polycyclic Aromatic Hydrocarbons
PBDE	Polybrominated Diphenyl Ethers
PCBs	Polychlorinated Biphenyls
PCDDs	Polychlorinated Dibenzo-p-dioxins
PCDFs	Polychlorinated Dibenzofurans
PFAS	Perfluoroalkyl substances
POPs	Persistent Organic Pollutants
SIR	Standardized Incidence Ratio
TCDD	2,3,7,8-Tetrachlorodibenzo-p-dioxin
UNEP	United Nations Environment Programme
WHO	World Health Organization

## References

[1] Stewart B.W., Wild C.P., World Health Organization/International Agency for Research on Cancer (WHO/IARC) (2014). Word Cancer Report.

[2] Bergman A., Jerrold J., Heindel J.J., Jobling S., Karen A., Kidd K.A., Zoeller R.T., World Health Organization/United Nations Environment Programme (WHO/UNEP) (2013). State of the Science of Endocrine Disrupting Chemicals 2012. An Assessment of the State of the Science of Endocrine Disruptors Prepared by a Group of Experts for the United Nations Environment Programme and World Health Organization.

[3] Solecki R., Kortenkamp A., Bergman Å., Chahoud I., Degen G.H., Dietrich D., Greim H., Håkansson H., Hass U., Husoy T. (2017). Scientific principles for the identification of endocrine-disrupting chemicals: Consensus statement. Arch. Toxicol..

[4] Vandenberg L.N., Colborn T., Hayes T.B., Heindel J.J., Jacobs D.R., Lee D.H., Shioda T., Soto A.M., vom Saal F.S., Welshons W.V. (2012). Hormones and endocrine-disrupting chemicals: Low-dose effects and nonmonotonic dose responses. Endocr. Rev..

[5] Kabir E.R., Rahman M.S., Rahman I. (2015). A review on endocrine disruptors and their possible impacts on human health. Environ. Toxicol. Pharmacol..

[6] Faniband M., Lindh C.H., Jönsson B.A. (2014). Human biological monitoring of suspected endocrine-disrupting compounds. Asian J. Androl..

[7] Soto A.M., Sonnenschein C. (2010). Environmental causes of cancer: Endocrine disruptors as carcinogens. Nat. Rev. Endocrinol..

[8] Morgan M., Deoraj A., Felty Q., Roy D. (2016). Environmental estrogen-like endocrine disrupting chemicals and breast cancer. Mol. Cell. Endocrinol..

[9] Liu R., Nelson D.O., Hurley S., Hertz A., Reynolds P. (2015). Residential exposure to estrogen disrupting hazardous air pollutants and breast cancer risk: The California Teachers Study. Epidemiology.

[10] Larsson S.C., Orsini N., Wolk A. (2015). Urinary cadmium concentration and risk of breast cancer: A systematic review and dose-response meta-analysis. Am. J. Epidemiol..

[11] Soto A.M., Sonnenschein C. (2015). Endocrine disruptors: DDT, endocrine disruption and breast cancer. Nat. Rev. Endocrinol..

[12] Aquino N.B., Sevigny M.B., Sabangan J., Louie M.C. (2012). Role of cadmium and nickel in estrogen receptor signaling and breast cancer: Metalloestrogens or not?. J. Environ. Sci. Health C Environ. Carcinog. Ecotoxicol. Rev..

[13] Sweeney M.F., Hasan N., Soto A.M., Sonnenschein C. (2015). Environmental endocrine disruptors: Effects on the human male reproductive system. Rev. Endocr. Metab. Disord..

[14] Giannandrea F., Paoli D., Figà-Talamanca I., Lombardo F., Lenzi A., Gandini L. (2013). Effect of endogenous and exogenous hormones on testicular cancer: The epidemiological evidence. Int. J. Dev. Biol..

[15] Paoli D., Giannandrea F., Gallo M., Turci R., Cattaruzza M.S., Lombardo F., Lenzi A., Gandini L. (2015). Exposure to polychlorinated biphenyls and hexachlorobenzene, semen quality and testicular cancer risk. J. Endocrinol. Investig..

[16] Prins G.S. (2008). Endocrine disruptors and prostate cancer risk. Endocr. Relat. Cancer.

[17] McGlynn K.A., Trabert B. (2012). Adolescent and adult risk factors for testicular cancer. Nat. Rev. Urol..

[18] Silva J.F., Mattos I.E., Luz L.L., Carmo C.N., Aydos R.D. (2016). Exposure to pesticides and prostate cancer: Systematic review of the literature. Rev. Environ. Health.

[19] Emeville E., Giusti A., Coumoul X., Thomé J.P., Blanchet P., Multigner L. (2015). Associations of plasma concentrations of dichlorodiphenyldichloroethylene and polychlorinated biphenyls with prostate cancer: A case-control study in Guadeloupe (French West Indies). Environ. Health Perspect..

[20] Béranger R., Le Cornet C., Schüz J., Fervers B. (2013). Occupational and environmental exposures associated with testicular germ cell tumors: Systematic review of prenatal and life-long exposures. PLoS ONE.

[21] The 2013 Berlaymont Declaration on Endocrine Disrupters. http://www.brunel.ac.uk/__data/assets/pdf_file/0005/300200/The_Berlaymont_Declaration_on_Endocrine_Disrupters.pdf.

[22] Pirastu R., Comba P., Conti S., Iavarone I., Fazzo L., Pasetto R., Zona A., Crocetti E., Ricci P. (2014). SENTIERI project—Epidemiological study of residents in national priority contaminated sites. Epidemiol. Prev..

[23] Orton F., Ermler S., Kugathas S., Rosivatz E., Scholze M., Kortenkamp A. (2014). Mixture effects at very low doses with combinations of anti-androgenic pesticides, antioxidants, industrial pollutant and chemicals used in personal care products. Toxicol. Appl. Pharmacol..

[24] Kortenkamp A. (2014). Low dose mixture effects of endocrine disrupters and their implications for regulatory thresholds in chemical risk assessment. Curr. Opin. Pharmacol..

[25] Zhu P., Liao L.Y., Zhao T.T., Mo X.M., Chen G.G., Liu Z.M. (2016). GPER/ERK&AKT/NF-κB pathway is involved in cadmium-induced proliferation, invasion and migration of GPER-positive thyroid cancer cells. Mol. Cell Endocrinol..

[26] Huang Y., Dong W., Li J., Zhang H., Shan Z., Teng W. (2014). Differential expression patterns and clinical significance of estrogen receptor-α and β in papillary thyroid carcinoma. BMC Cancer.

[27] He X., Jing Y., Wang J., Li K., Yang Q., Zhao Y., Li R., Ge J., Qiu X., Li G. (2015). Significant accumulation of persistent organic pollutants and dysregulation in multiple DNA damage repair pathways in the electronic-waste-exposed populations. Environ. Res..

[28] Lee J.C., Young-Ok Son Y.O., Pratheeshkumar P., Shi X. (2012). Oxidative stress and metal carcinogenesis. Free Radic. Biol. Med..

[29] Lin P.H., Lin C.H., Huang C.C., Chuang M.C., Lin P. (2007). 2,3,7,8-Tetrachlorodibenzo-p-dioxin (TCDD) induces oxidative stress, DNA strand breaks, and poly(ADP-ribose) polymerase-1 activation in human breast carcinoma cell lines. Toxicol. Lett..

[30] Silins I., Högberg J. (2011). Combined toxic exposures and human health: Biomarkers of exposure and effect. Int. J. Environ. Res. Public Health.

[31] Reaves D.K., Ginsburg E., Bang J.J., Fleming J.M. (2015). Persistent organic pollutants and obesity: Are they potential mechanisms for breast cancer promotion?. Endocr. Relat. Cancer.

[32] Crocetti E., Pirastu R., Buzzoni C., Minelli G., Manno V., Bruno C., Fazzo L., Iavarone I., Pasetto R., Ricci P. (2014). SENTIERI Project: Results. Epidemiol. Prev..

[33] International Agency for Research on Cancer (IARC) List of Classifications by Cancer Sites with Sufficient or Limited Evidence in Humans, Volumes 1 to 112. Last Update, 23 March 2015.

[34] Kortenkamp A., Evans R., Martin O., McKinlay R., Orton F., Rosivatz E., European Commission State of the Art Assessment of Endocrine Disruptors. Final Report. Annex 1. Revised Version, 29 January 2012.

[35] European Environment Agency The Impact of Endocrine Disrupters on Wildlife, People and Their Environments-the Weybrige+15 (1996–2011) Technical Report N. 2/2012.

[36] Gore A.C., Chappell V.A., Fenton S.E., Flaws J.A., Nadal A., Prins G.S., Toppari J., Zoeller R.T. (2015). EDC-2: The Endocrine Society’s second scientific statement on Endocrine-disrupting chemicals. Endocr. Rev..

[37] Musmeci L., Bellino M., Falleni F., Piccardi A. (2011). Environmental characterization of the National Contaminated Sites in SENTIERI project. Epidemiol. Prev..

[38] Beccaloni E., Cicero M.R., Falleni F., Piccardi A., Scaini F., Soggiu M.E., Vanni F., Carere M. (2014). Environmental characterization and exposure evaluation. Epidemiol. Prev..

[39] Miniero R., Ingelido A.M., Abballe A., di Domenico A., Valentinia S., Marra V., Barbieri P.G., Garattini S., Speziani F., De Felip E. (2017). Occupational exposure to PCDDs, PCDFs, and DL-PCBs in metallurgical plants of the Brescia (Lombardy Region, northern Italy) area. Chemosphere.

[40] Vimercati L., Baldassarre A., Gatti M.F., Gagliardi T., Serinelli M., De Maria L., Caputi A., Dirodi A.A., Galise I., Cuccaro F. (2016). Non-occupational exposure to heavy metals of the residents of an industrial area and biomonitoring. Environ. Monit. Assess..

[41] Ingelido A.M., Abate V., Abballe A., Albano F.L., Battista T., Carraro V., Conversano M., Corvetti R., De Luca S., Franchini S. (2016). Concentrations of polychlorinated dibenzodioxins, polychlorodibenzofurans, and polychlorobiphenyls in women of reproductive age in Italy: A human biomonitoring study. Int. J. Hyg. Environ. Health.

[42] Interdonato M., Bitto A., Pizzino G., Irrera N., Pallio G., Mecchio A., Cuspilici A., Minutoli L., Altavilla D., Squadrito F. (2014). Levels of heavy metals in adolescents living in the industrialised area of Milazzo-Valle del Mela (northern Sicily). J. Environ. Public Health.

[43] Bianco G., Zianni R., Anzillotta G., Palma A., Vitacco V., Scrano L., Cataldi T.R. (2013). Dibenzo-p-dioxins and dibenzofurans in human breast milk collected in the area of Taranto (Southern Italy): First case study. Anal. Bioanal. Chem..

[44] Vimercati L., Cuccaro F., Serinelli M., Bisceglia L., Galise I., Conversano M., Minerba S., Mincuzzi A., Martino T., Storelli M.A. Exposure assessment to heavy metals in general population in a polluted area through biological monitoring. Proceedings of the 16th International Conference on Heavy Metals in the Environment.

[45] Iavarone I., De Felip E., Ingelido A.M., Iacovella N., Abballe A., Valentini S., Marra V., Violante N., D’Ilio S., Senofonte O. (2012). Exploratory biomonitoring study among workers of livestock farms of the Taranto Province. Epidemiol. Prev..

[46] Esposito M., Serpe F.P., Neugebauer F., Cavallo S., Gallo P., Germana Colarusso G., Baldi L., Iovane G., Serpe L. (2010). Contamination levels and congener distribution of PCDDs, PCDFs and dioxin-like PCBs in buffalo’s milk from Caserta province (Italy). Chemosphere.

[47] Turrio-Baldassarri L., Abate V., Battistelli C.L., Carasi C., Casella M., Iacovella N., Indelicato A., La Rocca C., Scarcella C., Alivernini S. (2008). PCDD/F and PCB in human serum of differently exposed population groups of an Italian city. Chemosphere.

[48] Giandomenico S., Spada L., Annicchiarico C., Assennato G., Cardellicchio N., Ungaro N., Di Leo A. (2013). Chlorinated compounds and polybrominated diphenyl ethers (PBDEs) in mussels (*Mytilus galloprovincialis*) collected from Apulia Region coasts. Mar. Pollut. Bull..

[49] Matozzo V., Binelli A., Parolini M., Locatello L., Marina M.G. (2010). Biomarker responses and contamination levels in the clam *Ruditapes philippinarum* for biomonitoring the Lagoon of Venice (Italy). J. Environ. Monit..

[50] Grassi P., Fattore E., Generoso C., Fanelli R., Arvati M., Zuccato E. (2010). Polychlorobiphenyls (PCBs), polychlorinated dibenzo-p-dioxins (PCDDs) and dibenzofurans (PCDFs) in fruit and vegetables from an industrial area in northern Italy. Chemosphere.

[51] Turrio-Baldassarri L., Alivernini S., Carasi S., Casella M., Fuselli S., Iacovella N., Iamiceli A.L., La Rocca C., Scarcella C., Battistelli C.L. (2009). PCB, PCDD and PCDF contamination of food of animal origin as the effect of soil pollution and the cause of human exposure in Brescia. Chemosphere.

[52] Cardellicchio N., Buccolieri A., Giandomenico S., Lopez L., Pizzulli F., Spada L. (2007). Organic pollutants (PAHs, PCBs) in sediments from the Mar Piccolo in Taranto (Ionian Sea, Southern Italy). Mar. Pollut. Bull..

[53] American Public Health Association A Precautionary Approach to Reducing American Exposure to Endocrine Disrupting Chemicals. http://www.apha.org/policies-and-advocacy/public-health-policy-statements/policy-database/2014/07/09/09/03/a-precautionary-approach-to-reducing-american-exposure-to-endocrine-disrupting-chemicals/.

[54] European Environment Agency Late Lessons from Early Warnings: Science, Precaution, Innovation. EEA Report No 1/2013.

